# Sugar addiction: the state of the science

**DOI:** 10.1007/s00394-016-1229-6

**Published:** 2016-07-02

**Authors:** Margaret L. Westwater, Paul C. Fletcher, Hisham Ziauddeen

**Affiliations:** 1 Brain Mapping Unit, Department of Psychiatry, University of Cambridge, Cambridge, CB2 3EB UK; 2 Department of Psychiatry, Addenbrooke’s Hospital, University of Cambridge, Herchel Smith Building, Cambridge, CB2 0SZ UK; 3 Wellcome Trust MRC Institute of Metabolic Science, University of Cambridge, Cambridge Biomedical Campus, Cambridge, CB2 0QQ UK; 4 Cambridgeshire and Peterborough Foundation Trust, Cambridge, CB21 5EF UK; 5 Box 189, Herchel Smith Building, West Forvie Site, Robinson Way, Cambridge Biomedical Campus, Cambridge, CB21 5DS UK

**Keywords:** Sugar addiction, Obesity, Binge eating, Animal neuroscience, Drug addiction

## Abstract

**Purpose:**

As obesity rates continue to climb, the notion that overconsumption reflects an underlying ‘food addiction’ (FA) has become increasingly influential. An increasingly popular theory is that sugar acts as an addictive agent, eliciting neurobiological changes similar to those seen in drug addiction. In this paper, we review the evidence in support of sugar addiction.

**Methods:**

We reviewed the literature on food and sugar addiction and considered the evidence suggesting the addictiveness of highly processed foods, particularly those with high sugar content. We then examined the addictive potential of sugar by contrasting evidence from the animal and human neuroscience literature on drug and sugar addiction.

**Results:**

We find little evidence to support sugar addiction in humans, and findings from the animal literature suggest that addiction-like behaviours, such as bingeing, occur only in the context of intermittent access to sugar. These behaviours likely arise from intermittent access to sweet tasting or highly palatable foods, not the neurochemical effects of sugar.

**Conclusion:**

Given the lack of evidence supporting it, we argue against a premature incorporation of sugar addiction into the scientific literature and public policy recommendations.

## Introduction

Between 1980 and 2013, the proportion of overweight (body mass index (BMI) ≥ 25 kg/m^−2^) and obese (BMI ≥ 30 kg/m^−2^) adults rose from 28.8 to 36.9 % worldwide, with similar trends appearing in children and adolescents [1]. The accompanying costs of health consequences and absenteeism associated with excess weight, estimated to range from $3.38 to 6.38 billion annually in the USA alone, make obesity a pressing public health problem [2]. The scale and impact of the obesity pandemic are incontrovertible. The gravity of the situation demands extreme care and careful scrutiny of existing evidence rather than premature application of questionable concepts. In this spirit, we wish to evaluate sugar addiction because such a concept could have remarkable consequences in terms of public policy and health advice if generally accepted.

The food addiction (FA) model asserts that excessive consumption of palatable foods may be understood within the same neurobiological framework as drug addiction. The model of addiction here is that operationalised in the Diagnostic and Statistical Manual of Mental Disorders (DSM-IV) and one that is widely accepted in the neuroscientific literature. It is characterised by loss of control of drug consumption, increased motivation to consume, and a persistence of drug taking despite negative consequences, and the neurobiology of these behaviours has been extensively studied (see [3, 4]). Individuals who develop FA are thought to display symptoms analogous to those of drug abuse, including loss of control, withdrawal, and cravings for ‘problem foods’ [5]. Theron Randolph first used the term ‘food addiction’ in 1956 [6] to describe addictive-like consumption of various foods, such as corn, milk, eggs, and potatoes. However, aspects of the FA model have changed since this original description (see [7]), and there is an emerging view that highly processed foods, rich in sugar and fat, are most likely to be addictive. FA researchers argue that examining obesity through the lens of addiction will open new avenues for prevention, treatment, and public health policy [8, 9] though this, like many other aspects of the model, has been questioned [10, 11].

Sugar addiction represents a specific case of the FA model in which the addictive substance is the specific nutrient sugar. In this perspective article, we consider the state of the evidence in support of sugar addiction in humans and provide a critical review of the preclinical neuroscience research that has identified sugar addiction in rodent models. This is important because few studies have specifically examined sugar addiction in humans, and the bulk of supporting evidence comes from animal work. However, there is a methodological challenge in translating this work because humans rarely consume sugar in isolation. In order to assess the existing evidence, we must first consider whether sugar could be an addictive agent, examining specifically the animal neuroscientific evidence suggested to support this. As the animal neuroscience of sugar addiction draws strong parallels to drug addiction, we review the sugar and drug addiction neuroscience side by side. We go on to consider the human model of FA to determine whether and how it could be applied to sugar.

## Characterising (potentially) addictive foods

A general view is that FA is similar to substance addictions, rather than non-substance behavioural addictions outlined in the Diagnostic and Statistical Manual of Mental Disorders, 5th Edition (DSM-5, for a different perspective, see [12]), in that certain ‘addictive agents’ within food produce neurochemical effects in the brain similar to drugs of abuse. The Yale Food Addiction Scale (YFAS [13] and recently the YFAS 2.0 [14]), which is now the widely accepted measurement tool for studying FA, enquires about addiction-like eating behaviours with respect to ‘certain foods’. These scales do not specify nutrients of interest, yet this is only reasonable as we usually consume food with multiple nutrients. Even foods that may be predominantly composed of a single nutrient (e.g. sugar-sweetened beverages) have flavour(s) and other non-nutritive elements. However, examining the addictive potential of different foods may provide an indication as to whether any particular nutrient(s) are critical in determining addictive potential.

Evidence from rodent models supports high-fat [15], high-sugar [16], and, most strongly, combinations of high-fat and high-sugar [17] foods as candidates for FA. In humans, the FA construct extrapolates this view, surmising that highly processed, hyperpalatable foods are the ones that have addictive potential [18]. Lack of knowledge surrounding what might constitute an addictive food poses a substantial challenge, and to our knowledge, only two studies have examined the addictive potential of various foods.

Schulte et al. [5] suggest that certain highly processed foods share pharmacokinetic properties (inasmuch as the term can be used for food), such as high potency and rapid absorption rate, with drugs of abuse. The authors report that such processed foods are strongly associated with self-reported addictive eating as measured by the YFAS. Their findings also demonstrate that fat content and glycaemic load (GL, grams of carbohydrate per serving) predict ratings of problematic foods, where processed foods high in fat and/or GL are self-reported as more problematic. In this study, highly processed foods were defined as those high in fat and refined carbohydrates (high GL) that may also contain low levels of fibre, protein, and water content. Schulte et al. [5] argue that processing of raw foods increases the foods’ ‘potency’, or the absorption of the potential ‘addictive agents’ (e.g. fat, sugar, salt) into the bloodstream, as indexed by spikes in blood glucose levels following consumption.

Fowler et al. [19] hypothesised that individuals who developed substance use disorders post-bariatric surgery would be more likely to have had problems with foods that would cause high postprandial glucose levels. For this, they used foods listed in the YFAS and categorised them based on published glycaemic indices (GI), fat, and sugar content. Findings indicated increased likelihood of post-operative substance use amongst patients who endorsed high-GI and high-sugar, low-fat (but not high-sugar alone) foods as the most problematic [19]. Thus, the authors concluded that these patients might have experienced ‘addiction transfer’ resulting from previously undiagnosed FA. These findings should be interpreted in the light of several limitations. Apart from the retrospective recall of ‘problem foods’, only two foods (candy and soda pop) were classified as high sugar low fat. Furthermore, analyses of the relationship between problem foods and substance use onset failed to control for current or previous psychiatric morbidity, success of surgery, or current quality of life. Moreover, we would suggest caution in arguing that such foods are addictive based on the contentious concept of addiction transfer [20].

To describe the difference between foods such as cupcakes and bananas primarily as being one of the degrees of processing is perhaps a rather narrow view, and a strong case can be made for these foods having other important differences relevant to overconsumption and obesity (e.g. energy density). Even leaving this aside, there are several important concerns about both of these studies. First, the potentially addictive foods have been taken from the ‘problem foods’ list of the YFAS. The scale quantifies FA symptoms with respect to these problem foods, based on the assumption that they are likely to be addictive. Both of the aforementioned studies rely on this assumption and take the evidence that individuals have reported FA symptoms with respect to these foods on the YFAS in several studies, as further supporting the assumption. Second, these findings rely entirely upon participants’ perceptions of difficulties surrounding the foods items, which are then linked (by way of mechanistic explanation not empirical evidence) via GL or GI to postprandial glucose and insulin. That is, no direct evidence indicates that these foods are problematic for these individuals because they lead to higher postprandial glucose. Although individual postprandial glucose response (PPGR) has low intra-personal variability, there can be high interpersonal variability in PPGR following the consumption of identical meals [21, 22]. For example, Zeevi et al. [21] found that PPGRs for cookies and bananas varied significantly across participants, suggesting that some individuals may be high glucose responders to ‘good’ foods and low responders to ‘bad’ foods. It is also important to note that there are several high-GI foods such as breakfast cereals and baked potatoes that are not included in the list of YFAS problem foods. This suggests that the potential explanatory power of high GI as a determinant of addictive potential would lessen considerably if we looked beyond the list of problem foods. Given the host of metabolic, endocrine, and physiological factors that affect glucose regulation, it is important to consider the physiological response to foods as an interaction between the nutrient content of the food and the individual.

Third, and most importantly, the proposed model of why high-GI/high-GL foods may be potentially addictive lacks a mechanistic link between higher postprandial levels of glucose and addictive potential. Schulte et al. draw upon a seemingly superficial similarity between the addictive potential of drugs, based on their dose and speed of absorption, to explain why processed foods are likely to be addictive. Proponents of FA draw parallels between the processing of grapes to wine, poppies to opium, and the coca leaf to cocaine, which demonstrate the transition from naturally occurring substances/food to drugs of abuse and increasing potency via processing. Yet, this formulation of highly processed foods only captures the pharmacokinetic aspects of drugs of abuse, overlooking the critical pharmacodynamic effects. The coca leaf, for instance, has a pharmacodynamic effect, which can be enhanced by increasing the dose of the active ingredient through processing. For sugar or other foods, studies show that moderate increases in blood glucose following oral glucose ingestion can enhance cognitive performance in a variety of tasks, including semantic memory retrieval [23], reaction time tasks [24], and even driving performance [25]. Few functional MRI studies have examined the effect of blood glucose on brain function as it relates specifically to hedonic eating behaviours; however, Sun et al. [26] report that neither fasting nor postprandial blood glucose affected the blood-oxygen-dependent (BOLD) response to milkshake taste cues in several brain regions (e.g. amygdala, pallidum, insula) that have been implicated in drug cue studies. In men, increased postprandial blood glucose levels have been associated with increased resting state brain activity in regions associated with reward processing [27]. Given the tight physiological control over the stability of glucose supply to the brain, it is perhaps not unexpected that changes in systemic glucose would not have large effects on brain function. In short, the notion of increased dosage having increased potency and therefore increased addictive potential is questionable when it comes to sugar.

### Is sugar a potentially addictive substance?

The FA literature considers sugar (and other refined carbohydrates) to be a key facet of processed foods with high addictive potential, contributing to their GL (dose) and their rapid rate of absorption. Within this context, discussion of sugar has centred on its palatability or hedonic value; however, unlike substances of abuse, sugar has both hedonic and caloric value, and these two aspects broadly map onto ingestive and post-ingestive effects of its consumption, respectively. Moreover, these aspects are distinct and dissociable in terms of their neural processing as demonstrated in two elegant sets of experiments. Domingos et al. [28] showed that melanin-concentrating hormone (MCH)-expressing neurons located within the lateral hypothalamus respond to extracellular glucose levels and project to dopaminergic (DA) neurons in the striatum and midbrain regions. The animals show a preference for sucrose over the non-nutritive sweetener, sucralose, and the glucose-sensing ability of these neurons is critical in determining this, as transgenic mice lacking MCH neurons do not show this preference [28]. MCH neurons encode the rewarding nutrient properties of sucrose by increasing striatal DA release independently of gustatory input. Optogenetic stimulation of MCH neurons during consumption of sucralose leads to striatal DA efflux and preference for sucralose over sucrose [28].

Recently, Tellez et al. [29] expanded upon this work by examining DA transmission in the striatum in response to oral sucralose intake versus intra-gastric glucose or sucralose administration. Using microdialysis, the authors reported changes in DA release in the ventral and dorsal striatum, where regional DA release selectively encoded the pleasurable and nutritional value of the sweet foods. Sucralose consumption was linked to enhanced DA efflux in the ventral striatum (VS), which was no longer observed following devaluation of the sweetener with a bitter additive. Conversely, intra-gastric infusion of glucose, but not sucralose, elicited DA release in the dorsal striatum (DS). Thus, the VS and DS appear to encapsulate functionally distinct responses to palatable and nutritive signalling, and the authors went on to delineate the role of D1 and D2 striatal DA neurons in palatability and nutrient preferences. Dopaminergic signalling excites D1 DA neurons while inhibiting their D2 DA counterparts, and this interaction modulates the control of goal-directed actions, including overeating [17]. Optogenetic stimulation of D1 DA neurons within the DS and substantia nigra terminals increases consumption of a bitter sucrose solution, which supports the dorsal basal ganglia pathway as a circuit that is selectively responsive to the nutrient properties of sugar reward [29]. It should be noted, however, that the role of MCH, D1 DA, and D2 DA neurons has yet to be explored in animal models of sugar addiction, so whether the aforementioned neural circuits reflect processes underlying addictive-like sugar consumption remains unknown.

This experimental work allows us to consider that addictive-like properties of sugar may occur via three neural mechanisms: one related to palatability and the reinforcing effects of sweet taste, another related to caloric value and post-ingestive effects, and a third arising from a combination of the two effects. Put simply, the critical ‘addictive’ quality of sugar may be restricted to its sweetness, nutritional value, or some combination of the two. Of course, only the third possibility would support sugar as addictive, particularly within Schulte et al.’s model where highly processed foods with added sugar would be very sweet, energy dense, and rapidly absorbed and therefore potentially have a characteristic profile of ingestive and post-ingestive effects. Nonetheless, as humans often consume sugar in combination with other nutrients, differences between highly processed foods with high and low addictive potential would need to be characterised. Indeed, Zeevi et al. [21] demonstrated that the same foods can have very different post-ingestive profiles in different individuals. This may be a critical factor and one aspect of individual vulnerability to a potentially addictive food. These are theoretical considerations as thus far little work in humans has examined them directly. The animal literature does, however, offer some experimental evidence of parallels between sugar and drugs. We consider this in the next section, beginning with a brief overview of the neurobiological characteristics of drug addiction.

## Animal models of drug addiction

Prevailing models of drug addiction emphasise changes in reward-based learning and memory processes as core mechanisms involved in the transition from voluntary drug use to chronic abuse. Initially, goal-directed drug use releases DA within the mesolimbic system which reinforces ‘drug-taking’ behaviour by increasing the salience of, and subsequent motivation towards, drug-related cues [30]. Drug taking increases DA in the nucleus accumbens (NAcc) shell, yet this response becomes blunted over time in a manner that differs from habituation [31]. Instead, drug-related cues produce an anticipatory DA release in the DS, resulting in strong drug cravings [32]. This has been framed as an increased anticipatory reward with an attenuated consummatory reward. Activation in the dorsal striatum and basolateral amygdala drives ‘drug-seeking’ behaviour, and as this behaviour becomes increasingly elicited by drug-related cues, it is ultimately consolidated as a stimulus–response (S–R) habit [33]. This transition from goal-directed to habitual drug taking has been studied extensively (see [3, 34]) in rodent models of addiction to cocaine, heroin, and alcohol and strongly resembles compulsive drug use in humans. These compulsive behaviours arise from functional impairment in the prefrontal cortex (increased drug salience, compulsivity), as well as the dorsolateral and inferior cortices (compromised executive control) [35].

The onset of drug addiction has been associated with decreased availability of DA D2 receptors in both humans and non-human primates [36, 37]. These findings relate low DA receptor availability to increased trait vulnerability to drug abuse; however, it has been argued that chronic drug use reduces the number of DA D2 receptors, thus resulting in a ‘hypodopaminergic’ system [38]. While it is likely that aberrant DA D2 receptor numbers reflect both cause (trait vulnerability to) and consequence of prolonged drug use, reduced DA D2 receptor availability has been closely tied to withdrawal symptoms and the development of drug tolerance, in which drug consumption no longer elicits a positive effect but rather mitigates a negative state [39, 40]. Together with afferent input from the amygdala, these neuronal changes in the striatum (i.e. reduced DA D2 receptors) perpetuate drug use to avoid dysphoria and withdrawal, comprising what Koob and Le Moal [41] have termed the ‘dark side’ of addiction.

Accordingly, in sugar addiction, one could expect to see a similar behavioural and neurobiological syndrome. Voluntary consumption of sugar under goal-directed control would increase DA release in the mesolimbic system, enhancing the salience of and motivation for sugar. Over time, sugar seeking and consumption would become habitual and compulsive with an accompanying shift from ventral to dorsal striatal control, as well as changes in prefrontal cortical control of these behaviours. These neural adaptations would serve to perpetuate sugar seeking that may also be driven by the need to avoid withdrawal symptoms. In line with research of chronic drug use, DA D2 receptor levels may represent a vulnerability marker and also result as a consequence of excessive sugar intake over time, regardless of BMI status or obesity.

## Comparison of drug addiction and sugar addiction

Critical to these studies are the experimental designs used to model addiction-like behaviours in rodents. We believe that a working knowledge of these paradigms and their limitations is necessary to critically examine the literature on animal models of drug and sugar addiction. Thus, this section will provide an overview of common paradigms, and we will compare different aspects of drug and sugar addiction within this context. Comparisons have been drawn between sugar and a variety of illicit drugs, but for the purpose of this perspective article, we have chosen to focus on the neurobiological effects of cocaine, a stimulant that ‘hijacks’ the dopaminergic system, and heroin, an opiate that acts upon both dopaminergic and endogenous opioid systems. It is important to point out at the outset that sugar addiction literature is not as extensive as that of drug addiction literature, and therefore, not all aspects of addiction have been examined with respect to sugar.

### General overview of experimental models

#### Drugs

Rodent models of addiction traditionally frame the drug of choice as a positive reinforcer, which becomes associated with a pleasurable outcome. A drug is thought to function as a positive reinforcer if the animal’s response to the agent exceeds the response to a control, e.g. saline solution. Typically, animals are trained to self-administer the drug for a short daily session of 1 to 3 h [42] for 10 to 30 days [43]. For example, rodents may be trained to self-administer intravenous (IV) cocaine via a lever press or nose poke using a low ratio requirement where each lever press prompts drug delivery (a fixed ratio 1 (FR1) delivery). Drugs can be administered orally or intravenously, though it is often preferred to use the route most analogous to drug use in humans while taking into consideration taste effects. Thus, implanted catheters are usually used for IV infusion of cocaine and heroin, but some studies allow access to an oral cocaine–sucrose solution [44, 45]. It should be noted that, because many protocols train rodents to self-administer drugs of abuse prior to testing, this approach is insufficient to quantify vulnerability to drug addiction. As such, the use of drug-naïve animals has become increasingly commonplace.

To model the transition to compulsive ‘drug seeking’, the rodents are moved to progressive ratio (PR) tasks, in which they must systematically work harder (i.e. increase the number of lever presses for a single infusion). Motivation is further measured by ‘breakpoints’, or the ratio at which the animal is no longer willing to work for the reward, and it can be augmented by periods of drug abstinence. To examine the degree to which the animal will work for the drug despite negative consequences—a key feature of drug dependence—the conditioned stimuli (e.g. lever press) or outcomes are paired with aversive outcomes, such as an electric footshock or nauseating chemical additive. Following extensive drug self-administration, rodents display withdrawal symptoms in response to forced abstinence, as well as dopamine (e.g. sulpiride) and opioid (e.g. naloxone) antagonists. However, drug seeking can be extinguished throughout periods of forced deprivation by replacing the cocaine or heroin infusion with saline (for a complete review, see [46]).

In human addiction, habitual drug-seeking and drug-taking behaviour, even following sustained abstinence, is often elicited by environmental cues, acute stress, or drug exposure. Second-order reinforcement schedules represent one method by which cue-elicited reinstatement of drug seeking can be studied in animals [47]. The drug infusion is paired with an additional conditioned stimulus (e.g. illuminated light, tone) following which exposure to the conditioned stimulus has been shown to reinstate cocaine-seeking behaviour [48] and morphine administration [49] following abstinence. More recently, the conditioned place preference (CPP) paradigm has become a widely used design, in which rodents associate distinct environments with drug and saline infusions. Following abstinence, re-exposure to these environments, along with drug priming, leads to the reinstatement of habitual cocaine and heroin-seeking behaviour [50], thus modelling the circumstances under which humans often experience drug relapse.

#### Sugar

Although sugar (e.g. sucrose, saccharin, glucose) reinforcement has been widely used as a natural reward control within drug addiction studies, Avena et al. [16] have demonstrated that, under certain conditions, rats can develop addiction-like behaviours with respect to sugar. After over a decade of sugar addiction research, Hoebel et al. [51] claim to, ‘[…] still use the same basic technique to obtain clear signs of food dependency by imposing a feeding schedule that repeatedly induces sugar bingeing after a period of fasting’. In brief, this technique deprives rodents of food for 12 h (or in some instances, 16 h [52]) and permits free access to food for the subsequent 12 h, during which the rats may consume either chow or a sugar solution. Sugar is offered as either a 25 % glucose solution or a 10 % sucrose solution; the latter simulates a soft drink. For intermittent sugar access, the 12-h period of food availability begins 4 h into the dark cycle so as to increase rodents’ appetite and therefore the likelihood of consuming a novel food [51]. An important difference between the animals included in these experiments is that unlike the drug models, which increasingly use drug-naïve animals, these animals have usually had previous access to sucrose and are selected for sucrose preference (e.g. [53]). This raises the possibility of these animals having a vulnerability to developing this addiction-like syndrome. Rodents kept on this schedule for 3 to 4 weeks begin to develop signs of addiction, which we review below (see [54] for additional review). It is important to emphasise these addiction-like behaviours are only seen with sugar with intermittent access regimes and not with ad libitum access.

### Bingeing

#### Drugs

Following initial self-administration training, increased access (e.g. 6 h/day) to cocaine and heroin has been associated with enhanced, binge-like consumption [55–57]. Rodents with extended access to a low-dose cocaine infusion develop a binge-like pattern of consumption that increases rapidly at the outset, plateaus, and becomes highly variable after 24 h, where increased time between binges may serve to counteract the drug’s toxic effects [58]. Interestingly, binge-like self-administration of heroin may be moderated by satiety as food-restricted rodents self-administer the most heroin at the start of the session, but fed rodents self-administer heroin at a low, stable level throughout the session [57]. The reinforcing effects of both cocaine and heroin are dose dependent, and moderate doses have been shown to elicit reinforcing effects without leading to drug dependence [59].

Acute IV administration of cocaine preferentially increases extracellular DA in the NAcc shell when compared to the NAcc core [60], and this is associated with the acute reinforcing effects of cocaine. Heroin, too, increases DA release in both the ventral tegmental area (VTA) and the NAcc shell; however, this begins with the activation of mu-opioid receptors (MOR), which triggers a neurochemical cascade that leads to increased mesolimbic DA release [61, 62]. Mesolimbic DA release elicits hyperactivity and euphoric effects following cocaine and heroin infusion, respectively. These effects can be inhibited (as evidenced by reduced self-administration) by lesions to the ventral pallidum, as well as D1 receptor blockade in the central nucleus of the amygdala, in cocaine-conditioned animals [63, 64]. As heroin has high affinity for MOR and delta opioid receptors (DOR), administration of selective MOR and DA agonists has been shown to result in heroin reinforcement that is extinguished following chemical lesioning of DA neurons or microinjections of opioid receptor antagonists within the VTA [65].

#### Sugar

Binge-like sugar consumption has been observed in rodents under both 24-h and intermittent reinforcement schedules, where animals self-administer sugar on an FR1 protocol. Colantuoni et al. [66] reported that food-deprived rats increased sugar intake within the first hour of access to food, and similar bingeing patterns occur when rats receive 12-h access to both sugar and chow [51]. With the same intermittent reinforcement schedule, sham-fed rodents consume more sucrose than real-feeding controls [67], although differences are non-significant with repeated consumption. Interestingly, rodents with ad libitum access to sugar solution consume the food throughout the light phase (or inactive cycle), and total sugar intake does not differ between rodents with 12- versus 24-h access [16]. Moreover, rats fed daily intermittent sugar and chow offset sugar consumption by decreasing chow consumption, thus regulating caloric intake and preventing weight gain [68, 69]. Because rodents with ad libitum sugar access offset caloric intake and meal size throughout the testing period, Avena et al. [16] concluded that such experimental conditions cannot elicit sugar dependence. As such, it appears that the intermittent access is critical to the development of binging, as animals provided ad libitum access to sucrose fail to develop addictive behaviours. With respect to obesity, it is worth emphasising that rats on both intermittent and ad libitum access schedules offset chow intake to compensate for their sucrose intake and to maintain weight stability.

These behavioural data highlight noteworthy differences between sugar and drug bingeing. An immediately apparent distinction arises from temporal discrepancies related to forced deprivation of sugar versus drugs of abuse. Despite limited evidence of food restriction increasing vulnerability to chronic cocaine use [70], rodents increase *both* cocaine and heroin intake under normal feeding conditions, or those which maintain rodents at 85 % body weight (e.g. [71]). Under such conditions, it is possible to delineate the reinforcing effects of drugs of abuse versus non-drug rewards; however, these processes become conflated when sugar is only presented following food restriction. As similar findings are seen in sham-fed rats, it suggests that sugar bingeing results from the reinforcing effects of a preferred flavour, rather than post-ingestive effects of sucrose [54]. Under ad libitum conditions, rats dramatically increase cocaine intake initially, and, although bingeing becomes variable, rats continue to binge throughout the 72-h period [58]. Minimal restriction of cocaine self-administration has led to bingeing patterns that converge with an inherent circadian rhythm, as rodents repeatedly refused to self-administer cocaine during the light phase [72]. Yet, binge-like consumption of sugar appears to follow a distinct consummatory pattern with binges occurring early in the food available period, which likely arises from both homoeostatic regulation of feeding behaviour and the presence of palatable food.

The neurobiology of sucrose reinforcement has largely focused on dopaminergic effects in the NAcc shell and core. Intermittent sucrose consumption persistently increases extracellular DA in the NAcc shell and core in response to sugar in both sham [67] and normal feeding [16, 52] schedules. This effect does not appear in either control or ad libitum sugar access animals, and as with most foods, the DA response to sugar quickly habituates [73, 74]. Thus, a drug-like DA response to sugar is only observed in the intermittent binging paradigm, suggesting a critical role of the paradigm. Corwin has raised the possibility that this paradigm promotes a form of eating under uncertainty because food availability is unpredictable [75, 76].

Infusion of a selective mu-opioid agonist into the NAcc has led to increased consumption of sweet foods (e.g. chocolate) with identical nutrient profiles, suggesting that increased mu-opioid receptor binding underpins flavour rather than sucrose preference [77]. Additionally, MOR agonism in the NAcc has enhanced saccharin intake [78]. Infusion of naltrexone (an opioid antagonist with high MOR affinity) directly into the NAcc decreased consumption of the preferred flavour, yet systemic injection decreased consumption of both foods equally. These findings, along with those of Tellez et al., demonstrate distinct neural mechanisms for sweetness and caloric content, and support the role of rewarding effects of sweet taste in this intermittent access paradigm. Benton [54] and Dileone et al. [79] have previously argued the post-ingestive properties of glucose appear to have little effect on initial consolidation of its rewarding properties. Moreover, neurobiological changes in the striatum have yet to be reported in the absence of the intermittent sugar binging (i.e. with ad libitum access to sugar) [66]. In summary, the dopaminergic changes that resemble addiction only occur with sugar consumption under the intermittent access regime, and without these conditions, the dopaminergic response to sugar resembles that to other natural rewards. Conversely, cocaine and opiate drugs cause neurobiological changes within the NAcc and VS that lead to and perpetuate addiction, including changes in D2 DA receptor levels [3] and MOR density and expression [80] following chronic cocaine and opiate administration, respectively.

### Motivation and substance seeking

#### Drugs

Following initial self-administration training, rodents show increased motivation for cocaine self-administration as evidenced by high breakpoints within PR schedules. Breakpoints may be manipulated by several experimental parameters, including the unit injection dose and restricted access to cocaine. For example, rats that were allowed access to cocaine 4 times/h in a 24-h period during initial self-administration showed higher breakpoints after 7 days of abstinence when compared to rats that were initially allowed 72-h access [81]. Roberts et al. [55] assert that a progressive increase in daily breakpoints is not only dose dependent, but also moderated by the speed of the injection. For example, in rodents with a history of cocaine use, animals that received 3.0 mg/kg doses had significantly higher breakpoints than those that received 1.5 mg/kg doses [82]. Over several days of testing, speed of initial cocaine infusion significantly altered breakpoints, with higher breakpoints observed in rodents receiving cocaine infusions over 5 s versus those receiving slower infusions (e.g. 25 or 50 s) [83].

Unlike cocaine seeking, the emergence of heroin-seeking behaviour is closely tied to the onset of acute withdrawal symptoms, which result in increased consumption by way of negative reinforcement (i.e. avoidance of a dysphoric state). Acute opiate exposure increases pain sensitivity, which worsens with chronic use, and sensitisation of nociceptive systems may be related to the development of drug dependence via negative reinforcement [57, 84]. Both forced deprivation and opioid antagonists produce a withdrawal syndrome characterised by teeth chattering, paw tremors, and erratic activity [80].

Cocaine abstinence increases motivation in rodents initially trained on PR but not FR schedules, suggesting that the establishment of cocaine as a positive reinforcer powerfully enhances drug seeking after abstinence [85]. Moreover, Vanderschuren and Everitt [71] demonstrated that presentation of an aversive footshock does not suppress cocaine seeking in rodents with a prolonged cocaine self-administration history. Importantly, the authors assessed drug-seeking behaviour within a heterogeneous seeking–taking chain schedule, in which seeking and taking cocaine are distinct acts with separate levers. Additionally, pairing both cocaine–sucrose and lemon–sucrose solutions with an aversive lithium chloride injection has been shown to only devalue the lemon–sucrose solution as rodents maintained the same level of drug seeking for the cocaine solution [45].

Changes in the limbic, cortical, and ventral striatal circuitry mediate the development of drug-seeking behaviour [34]. Lesioning of either dopaminergic circuitry in the basolateral amygdala or glutamatergic circuitry in the NAcc core alters cocaine seeking [86]. In contrast, lesioning of medial PFC subregions enhances cocaine seeking [87], likely by way of diminished executive control, as this region projects to the posterior dorsomedial striatum (pDMS) and reciprocally to the basolateral amygdala [34]. The DA D2 system appears central to the development of enhanced motivation for morphine. Mice lacking D2 DA receptors equally pursue morphine and saline infusions on FR or PR schedules [88]; however, rodents with increased proenkephalin gene expression in the NAcc and DS demonstrate significantly higher breakpoints for morphine infusion than wild-type animals [89]. Thus, converging neurobiological evidence identifies both dopaminergic and opioid systems in the maintenance of opiate seeking. Over time, these neurobiological changes lead to the loss of control over drug seeking and intake, resulting in the hallmark feature of addiction—habitual drug seeking.

#### Sugar

Enhanced motivation and sugar seeking are often achieved by forced deprivation, which has increased the number of lever presses for self-administration of sucrose solution [16]. However, these findings do not directly represent rodents’ motivation for a sugar reward but rather the number of unsuccessful lever presses executed under an FR1 schedule (i.e. the lever presses in between sugar receipt). Receipt of sugar reward was not dependent upon the number of additional presses between reinforcement. A more recent study has incorporated differential reinforcement schedules, which systematically increase the time intervals between sucrose reinforcements to quantify impulsive responding for sucrose solutions [90]; however, the findings failed to demonstrate increased lever pressing across sucrose-reinforced sessions as compared to control (i.e. water) sessions. As such, motivation for sucrose appears to be less robust than that for either cocaine or heroin, though expectedly infusion of a selective mu-opioid agonist significantly increases break points for sugar pellets in a progressive ratio schedule [91].

Some research has quantified motivation for sucrose by direct comparison with other drug-seeking behaviours. In one study, some rodents preferred self-administration of saccharin over cocaine and paid a greater ‘price’ for saccharin than for cocaine by adhering to FR2, FR4, and FR8 reinforcement schedules [53]. Although this resembles early PR schedules in which rodents linearly increased lever presses for subsequent infusions, standard PR schedules for drug reinforcement now require rats to increase lever presses exponentially from one infusion to the next [55]. Thus, direct comparison of these findings to those from PR schedules of cocaine and heroin reinforcement overestimates the degree to which saccharin increases motivation. Rodents bred for high-saccharin selectivity increased cocaine consumption following reinstatement of drug-seeking behaviour, yet the effect(s) of sweet preference on vulnerability to drug addiction remain poorly understood [92]. For example, preference for Oreo cookies has predicted greater break points on a PR schedule for IV cocaine infusion, yet rodents that preferred rice cakes demonstrated equivalent self-administration, tolerance, and reinstatement of cocaine-seeking behaviour [93].

### Habitual use and withdrawal

#### Drugs

Rodents with extended cocaine self-administration training preferentially return to environments in which cocaine was administered, even following periods of abstinence (see [94, 95]). Exposure to the conditioned stimulus (i.e. a light previously paired with lever pressing) has been shown to reinstate cocaine-seeking behaviour [48] and morphine administration [49] following abstinence. A combination of drug priming, or drug injections following abstinence, and the CPP paradigm restores habitual cocaine and heroin [50]-seeking behaviour, thus modelling the circumstances under which humans often experience drug relapse.

Whereas the acute reinforcing effects of cocaine are associated with increased extracellular DA in the VS and NAcc shell, cocaine seeking has been related to enhanced DA in the DS independent of the NAcc [96]. Blockade of DA receptors in the anterior dorsolateral striatum, but not the pDMS or NAcc, decreases drug seeking [97]. Jedynak et al. [98] further demonstrated that prolonged stimulant use alters synaptic connectivity in DS neurons by increasing dendritic spine density in the dorsolateral subregion and decreasing spine density in the dorsomedial subregion. The authors assert that such restructuring of synaptic connectivity in the DS underlies the emergence of S–R habits following chronic stimulant use as the dorsolateral striatum gains control of these behaviours. As discussed above, in the case of heroin, the opponent processes model describes the persistence of drug use as negatively reinforced by the dysphoria of withdrawal symptoms [99].

#### Sugar

Although compulsive sugar-seeking behaviour following extended consumption has yet to be studied explicitly, converging evidence suggests that animals develop CPP in response to food rewards. After abstaining from sugar, food-deprived rodents prefer the environments in which 12 and 20 % sucrose solutions were consumed [100, 101], and similar findings were reported with high-sucrose food rewards [102]. Administration of naltrexone dose-dependently disrupts CPP for sucrose, yet the opioid antagonist does not affect the development of CPP [103]. The competitive opioid antagonist naloxone precipitates withdrawal symptoms in sugar-bingeing rats, which resemble those of opiate withdrawal (e.g. anxiety, teeth chattering, forepaw tremor, head shakes) and share a similar neural profile with decreased DA and increased acetylcholine in NAcc [66]. Furthermore, Avena et al. [104] report increased anxiety in fasted rodents (36 h) that were previously maintained on an extended intermittent reinforcement schedule with 10 % sucrose solution. A similar withdrawal syndrome has been observed following 8 days of an intermittent access to saccharin [51]. It has also been demonstrated that rats on the intermittent access schedule show reduced D2 DA receptor binding in the DS [66].

### A shared neurobiology?

An oft-repeated observation asserts that food and drug consumption share a common neurobiology [105]. This is true in so far as drugs are understood to ‘hijack’ a neural system that primarily processes natural rewards like foods; however, important differences remain. First is the matter of the anatomical localisation of the neural circuits involved in these consummatory behaviours. Carelli et al. [106] have demonstrated that different populations of neurons in the NAcc respond to cocaine and natural rewards. Second, the dopaminergic response to sugar (and other foods) rapidly habituates, and it is attenuated by predictive cues such as smells; however, the DA response to cocaine does not habituate and is enhanced by predictive cues [31]. Third, when cue pairing to the delivery of either sugar or cocaine is established, the cue results in a dopaminergic surge. Importantly, in the case of sucrose, the DA level rapidly returns to baseline and does not rise again with lever pressing or consumption of sucrose [107] whereas in cocaine, the surge does not return to baseline but further increases after lever pressing and cocaine delivery [108]. Fourth, Pavlovian stimuli conditioned to food release DA in the NAcc core, whereas those conditioned to drugs of abuse release DA in the shell [54, 109].

### Summary of the animal neuroscience

Clearly, addiction-like behaviours can be elicited by sucrose, but there are two important caveats to bear in mind. First, as evidenced by the studies using sucrose in sham-fed animals, and those that used real feeding with saccharin, it seems that these behaviours occur in response to the palatability of sweet tastants, not the caloric content. Both of these findings raise another important question: Are there any pharmacodynamic effects of sucrose that are important to the development of this addiction syndrome, in the way that pharmacodynamic effects of drugs are critical to the development of the neuroadaptive changes in addiction? Second, these behaviours are only engendered in a specific intermittent access regime, which seems critical to their development, as these behaviours are not seen in animals given ad libitum access to sugar. Moreover, within this regime, test animals have been pre-selected for sucrose preference. This practice has become largely obsolete in animal models of drug addiction where drug-naïve animals are preferable. By excluding sucrose-naïve animals, the prevalence of addictive-like sucrose consumption remains unknown as opposed to cocaine or heroin addiction, where it has been estimated that between 5 and 24 % of individuals who use drugs go on to develop drug addiction [110–112]. Clearly, the combination of sweet taste and intermittent access can trigger a state that strongly resembles addiction in several aspects, including a cross-sensitisation effect to amphetamine and alcohol [68, 113] that seems to be mediated by mu-opioid receptor binding.

However, even in the intermittent access model, there remain several key deficiencies in the case for a sugar addiction. To date, increased motivation for sucrose has been poorly modelled because few studies have implemented progressive ratio schedules to measure the rodents’ willingness to work for sugar. Rodents with extended access to sugar remain susceptible to devaluation procedures, such as the addition of a nausea-inducing agent, whereas cocaine- or heroin-addicted animals continue to pursue the drug despite negative consequences. The extent of habitual responding to sugar remains understudied, and the effect of CPP on reinstatement of sucrose seeking has yet to be characterised. In contrast, the presentation of conditioned stimuli reliably reinstates drug-seeking behaviours in animals with historic cocaine or heroin use, and the reinstatement of habitual drug seeking in response to environmental cues represents a hallmark feature of addiction. Taken together, addictive-like consumption of sugar diverges from drug addiction on both neurobiological and behavioural levels, suggesting a need for great caution in drawing parallels between sugar and drug addiction.

## Sugar addiction in humans

There has been little empirical work examining sugar addiction in humans. Given this, we consider how sugar addiction, as a specific form of FA, might be conceptualised in humans, and we summarise experimental challenges in evaluating it, beginning with a brief overview of FA.

### The behavioural phenotype of food addiction: the YFAS and YFAS 2.0

The current FA phenotype was first operationalised in the 25-item Yale Food Addiction Scale (YFAS; [13]). Both the FA model and the YFAS conceptualised FA in terms of a translation of DSM-IV substance dependence [114] to food. Criteria include persistent eating despite negative consequences, persistent desire for food, unsuccessful attempts to cut down and impairment of functioning because of overeating. The criteria are defined with respect to ‘certain foods’, and the YFAS provides 21 examples from 5 food categories: sweets (e.g. ice cream), starches (French fries), salty snacks (pretzels), fatty foods (pizzas), and sugary drinks. The YFAS can provide a ‘diagnosis’ of FA if at least three criteria are endorsed along with clinical impairment, or a ‘symptom count’ to indicate severity of symptomatology (scores range from 0 to 7). It has become a popular and widely used self-report measure of this construct to the extent that it is used to both define and measure FA, though its validity and utility have been questioned [10, 11].

The YFAS has recently been updated [14] based on DSM-5 criteria for substance-related and addictive disorders in the YFAS 2.0. The key difference is that, in updating criteria according to DSM-5, which incorporates both abuse and dependence, the threshold for diagnosing FA has been reduced. As such, individuals experiencing clinically significant impairment may be diagnosed with mild (2–3 symptoms), moderate (4–5 symptoms), or severe (6 or more symptoms) FA. Preliminary validation of the YFAS 2.0 estimates that 15.8 % of individuals meet criteria for FA, and 11.9 % of the sample met the threshold for severe FA. As with the YFAS, overweight and obese individuals endorsed more FA symptoms than their lean counterparts, and the prevalence rate of severe FA was highest in the obese weight class [14]. Although the authors report improved internal consistency and convergent validity in the YFAS 2.0, previously expressed concerns (see [11, 115]) regarding the inclusion of withdrawal symptoms and tolerance and how they might relate to foods remain. With respect to these, the main concern is not that their presence is critical in FA; rather, their inclusion in the scale is undermined by the fact that they are not adequately defined and may therefore mean different things to different respondents. Moreover, withdrawal is frequently endorsed by participants in several YFAS studies, and from the development study of the YFAS 2.0, there seems to be strong concordance between the withdrawal items in the YFAS and the YFAS 2.0 [14]. It is important, therefore, that they are characterised clearly and rigorously. Given the lack of precise definition, it is difficult to determine conclusively that endorsement of this item reflects withdrawal symptoms to a particular nutrient or food. Indeed, if a withdrawal syndrome could be rigorously characterised, it would offer important clues as to the nature and mechanism of action of the addictive substance. However, here it is important to acknowledge the difficulty posed by the lack of a clearly defined addictive agent or food.

### Does food addiction represent a distinct phenotype?

FA has several shared features and high levels of co-morbidity with binge eating disorder (BED) [10, 116], which raises the question: could it be that YFAS is indirectly measuring a syndrome already well described as opposed to defining a distinct syndrome? BED is characterised by the consumption of objectively large portions of food with loss of control over eating, which is often done in isolation and followed by feelings of guilt and disgust. It is associated with weight gain, but a significant proportion of people with BED are not obese. Patients with BED have been proposed to be the strongest candidates for FA [117], and some researchers have proposed that FA represents an atypical subtype of BED based on a growing body of the literature that has identified shared genetic vulnerabilities to drug abuse and binge eating. Others have suggested that individuals with BED exhibit poor impulse control and emotion regulation, as well as aberrant reward processing, which may increase FA liability [118]. Davis et al. [119] found that BED was associated with the A118 polymorphism of the mu-opioid receptor gene (*OPRM1*) and the Taq1A A1 polymorphism of the dopamine D2 receptor gene (*DRD2*), both risk factors for substance use disorder. This same group also identified a dopaminergic multilocus genetic profile that is uniquely associated with FA when controlling for binge eating behaviours [120]. These data suggest a similarity between FA and substance addictions, but require further exploration in well-powered studies with the appropriate diagnostic groups is necessary.

Long et al. [116] recently carried out the first systematic review of the YFAS literature. They examined 40 published articles to address important outstanding questions about FA, including its relationship with BMI and eating disorder pathology and whether FA represents a distinct phenotype of disordered eating. The authors found a high co-occurrence of FA with BED and bulimia nervosa. An estimated 47.2 to 56.8 % of people with BED meet criteria for a FA ‘diagnosis’ [116], and these prevalence rates seem excessive for a diagnostic subgroup. Binge eating frequency correlated with YFAS scores in both overweight and healthy weight groups, but the relationship with BMI was less clear-cut. Some studies report non-significant differences in BMI across YFAS-diagnosed ‘food addicts’ and their healthy counterparts [121], while others indicate no correlation between BMI and YFAS score [122, 123]. While the prevalence rates of FA are consistently greater in overweight and obese groups (15.2 to 56.8 %), whether FA accounts for enough unique variance in obesity to be considered an explanatory mechanism for this condition remains unclear. Furthermore, the highest prevalence rates of FA have been reported in individuals with bulimia nervosa (83.6 %) [124, 125]. This finding should be interpreted cautiously as the numbers of individuals with diagnosed bulimia nervosa in these studies is small. Nevertheless, as these individuals often maintain a healthy BMI, it remains plausible that FA prevalence could be dissociable from BMI, particularly amongst those who have distorted thoughts related to food consumption. In summary, the findings of Long et al. [116] provide evidence of significant heterogeneity in the behavioural correlates of FA and suggest poor discriminant validity of the YFAS.

### Defining a sugar addiction in humans

Defining sugar addiction in humans remains challenging. First, as we have discussed earlier, little evidence supports sugar as an addictive substance, and the animal neuroscience literature suggests sweetness or palatability to be critical elements of addictive-like eating. That is, sweet foods rather than sugar per se might be the ‘substance’ of interest. Even so, there remain important questions about how sweetness or sugar content relates to addictive potential and whether sugar is necessary. Second, current measurement of FA is insufficiently precise, and given a commonplace behaviour like consumption of sweet food, it will be critical to define a profile of consumption that separates normal from disordered intake. The YFAS attempts to do this by using severity criteria for individual items and a necessary overall impairment criterion for diagnosis [13]. Although preliminary, examination of dietary profiles associated with problematic eating in young adults has shown that consumption of energy-dense, nutrient-poor foods (e.g. candy, take out meals) is positively correlated with FA score and BMI [126]. Interestingly, dietary intake of carbohydrates or sugar was not significantly associated with FA diagnoses or scores, suggesting a limited role of sugar in putative addictive-like eating in humans. Third, whether FA represents a distinct phenotype remains unclear, and the high degree of diagnostic overlap with BED is a particular difficulty. Distinguishing individuals with BED who preferentially binge on sweet foods from those with a sugar (or sweet food) addiction will be a challenging yet critical step towards a more refined FA phenotype.

An alternative approach would be to consider whether aspects of sugar or sweet food consumption share a similarity with addiction-like behaviours, such as cravings (for a review, see [54]). The general population often reports food cravings, particularly for palatable foods like chocolate. However, these cravings differ from drug cravings in terms of their intensity, their reported frequency and/or their duration. Food cravings are relatively short-lived and subside with fasting as opposed to drug cravings, which persist and do not lessen in intensity with abstinence [54, 127]. Rogers and Smit [127] have proposed an alternative formulation: seeing food cravings in terms of ambivalent attitudes to particular foods. Thus, for some people, chocolate is a highly desirable food but one that should be eaten with restraint. Attempts to restrain intake make chocolate more salient and preoccupying, and this is experienced as a craving and hence, perhaps, likened to an addiction. In part, this alternative approach asks whether there is an addictive aspect to normal eating (of sweet foods), and this is highly debatable.

## Conclusions

In this perspective article, we have reviewed the current state of the evidence for sugar addiction. Most of the evidence is limited to the animal neuroscience literature, and it is far from convincing. Importantly, several key elements of drug addiction have not been evaluated in sugar addiction models, such as the transition to compulsive drug-taking and dose-dependent effects on addiction liability. There remains a paucity of human evidence in this area, and we did not consider the literature encompassing the behavioural and neural effects of sweet or palatable food consumption as this would be far too indirect to the question of sugar addiction. There is the problem of the dearth of data on pure sugar consumption as we rarely consume sugar in isolation, and the ecological validity of studies examining pure sugar consumption in humans would be limited.

In terms of future directions, we suggest two areas of potential interest. The first is to examine whether sweet foods with high GI/GL might cause a food addiction in humans. We have discussed the significant methodological and conceptual limitations of the human FA model and its measurement instruments, the YFAS and the YFAS 2.0, which will need to be considered in such explorations. The second is to examine the relevance of the intermittent sugar access schedule used in animal models to the development of eating disorders (and perhaps even a form of FA) in humans.

In summary, the science of sugar addiction at present is not compelling. Nevertheless, sugar addiction remains a very popular and powerful idea, but as this special issue illustrates, it is by no means alone in this regard when it comes to misconceptions about sugar. Even the most perfunctory Internet search reveals how much emotive and explanatory power the term ‘sugar addiction’ has when used in its lay sense for individuals personally, as well as in the context of major public debates such as those over the sugar tax or campaigns such as Action on Sugar in the UK. Although the concept as we discuss it here is far more rigorous, the lay interpretation raises the question of whether sugar addiction is a useful (if not valid) concept to help tackle obesity and/or change the food environment? From a policy perspective, it is unlikely that sugar could be excluded from individuals’ diets given its presence in numerous food items, and any analogies suggested based on the regulation of illicit drugs would be specious. Given the multitude of interacting factors that increase one’s risk for eating disorders and obesity, we argue that support of sugar addiction as a primary causal mechanism of weight gain represents an extremely narrow view that fails to capture the complexity of these conditions, and one that may hamper more coordinated and appropriate responses. Furthermore, while there is a pressing need to address these important concerns, we argue that it is dangerous to draw strong conclusions about the validity of sugar addiction based on the current evidence. There are many strong arguments for cutting down the consumption of sugar and reformulating food products accordingly, yet these arguments will all stand or fall according to the scientific case that supports them.
